# The role of smoke from cooking indoors over an open flame and parental smoking on the risk of cleft lip and palate: A case- control study in 7 low-resource countries

**DOI:** 10.7189/jogh.10.020410

**Published:** 2020-12

**Authors:** Allyn Auslander, Roberta McKean-Cowdin, Frederick Brindopke, Beau Sylvester, Melissa DiBona, Kathy Magee, Rijuta Kapoor, David V Conti, Sylvia Rakotoarison, William Magee, Operation Smile International Family Study Group

**Affiliations:** 1 Department of Preventive Medicine, Keck School of Medicine of the University of Southern California, California, USA; 2 USC Eye Institute, Department of Ophthalmology, Keck School of Medicine of the University of Southern California, California, USA; 3 Children’s Hospital Los Angeles, Los Angeles, California, USA; 4 Operation Smile, Inc.; Virginia Beach, Virginia, USA; 5 Operation Smile Madagascar; Antananarivo, Madagascar, USA

## Abstract

**Background:**

Cleft is one of the most common birth defects globally and the lack of access to surgery means millions are living untreated. Smoke exposure from cooking occurs infrequently in developed countries but represents a high-proportion of smoke exposure in less-developed regions. We aimed to study if smoke exposure from cooking is associated with an increased risk in cleft, while accounting for other smoke sources.

**Methods:**

We conducted a population-sampled case-control study of children with cleft lip and/or palate and healthy newborns from Vietnam, Philippines, Honduras, Nicaragua, Morocco, Congo, and Madagascar. Multivariable regression models were used to assess associations between maternal cooking during pregnancy, parental smoking, and household tobacco smoke with cleft.

**Results:**

2137 cases and 2014 controls recruited between 2012-2017 were included. While maternal smoking was uncommon (<1%), 58.3% case and 36.1% control mothers cooked over an open fire inside. Children whose mothers reported cook smoke exposure were 49% (95% confidence interval (CI) = 1.2-1.8) more likely to have a child with a cleft. This was consistent in five of seven countries. No significant associations were found for any other smoke exposure.

**Conclusions:**

Our finding of maternal cook smoke and cleft in low-resource countries, similar to maternal tobacco smoke in high-resource countries, may reflect a common etiology. This relationship was present across geographically diverse countries with variable socioeconomic statuses and access to care. Exposures specific to low-resource settings must be considered to develop public health strategies that address the populations at increased risk of living with cleft and inform the mechanisms leading to cleft development.

Surgically treatable conditions account for approximately one-third of all global disease and an additional 2.2 million providers would be needed to treat the existing surgical need [1]. Although such conditions are treatable, many impacted children and adults do not have adequate access to care. Prevention of surgically treatable birth defects is therefore a necessary goal as provision of surgical treatment for all patients is unlikely - especially for diseases such as orofacial clefts that require complex multidisciplinary care.

Cleft lip with or without palate is one of the most common birth defects worldwide. The global incidence is approximately 1 in 700 live births [2], however incidence ranges from 1.28-1.90 per 1000 live births in Asians, 0.96-1.87 per 1000 in Hispanics, 1 per 1000 in non-Hispanic Whites, and 0.73-1.22 per 1000 in populations of African descent [2-5]. Although there is clear variability by ethnicity, the majority of etiologic data comes from individuals of European descent [6]. Disruptions in craniofacial development occur during the first trimester of pregnancy during weeks 4 to 13 of development [7]. Cleft is characterized as an embryologic failure of fusion of facial elements that normally develop into the lip and palate. In the absence of any other birth defect, the patient is considered to be non-syndromic (approximately 70% of cleft lip with or without palate and 50% of isolated cleft palate patients) [2,6-8]. While the origins of syndromic clefts are considered largely genetic, the etiology of non-syndromic clefts remains unclear.

Parental smoking has been considered an important determinant of developmental disorders, but the environmental impact of smoke exposure from cooking and cleft risk has only been mentioned in two existing studies [9,10]. In low-resource countries, biomass cooking fuel is used by approximately 80% of rural households and has been associated with a wide variety of diseases, including stillbirths [11]. While maternal smoking has been associated with risk of cleft [12-14], the association between smoke from cooking remains uncharacterized. Beyond different types of smoke exposure, the main established risk factors for cleft are low maternal education [15,16], lack of folic acid supplementation [17,18], advanced maternal age [19,20], family history of clefts [21], and ethnicity [22]. Other factors that have been less consistently associated with cleft are periconceptional alcohol use [23,24] and diabetes (either pregestational or gestational) [25,26].

In the current analysis, we used data from over 4000 children and their mothers collected on surgical missions conducted by Operation Smile. Specifically, we assessed the relationship between smoke exposure from cooking and the risk of non-syndromic cleft. Other sources of smoke investigated include maternal smoking, paternal smoking, and household tobacco smoke. This study is the first to evaluate cooking practices as an environmental determinant of cleft in a population-recruited sample of children from diverse, low-resource countries. Data were collected from 7 countries (Vietnam, Philippines, Morocco, Madagascar, Democratic Republic of Congo (DRC), Honduras, Nicaragua) to evaluate the association overall and to explore factors that may influence heterogeneity of effects by country. Clarifying the role of prenatal exposure to smoke from sources common to different populations and cleft risk may help to improve our understanding of risk factors contributing to non-syndromic cleft and inform preventive strategies.

## METHODS

Data for this study was collected from 2012-2017 as part of a coordinated series of population-sampled case-control studies focusing on genetic, lifestyle and environmental exposures and cleft in children 6 months to 4 years of age. This study was conducted with Operation Smile (OS), an internationally recognized not-for-profit that has been providing free cleft surgery and related care to patients for over 36 years. Data for the current analysis represents children from 7 countries sampled over multiple missions (Table S1 in the **Online Supplementary Document**). Participation rates in the study varied by site from 77%-96% for cases and 45%-100% for controls. The methods of this study have been previously published in depth [25,27]. All work was approved by the Institutional Review Board at the University of Southern California including country-specific authorizations.

### Case definition

This study includes non-syndromic cases of cleft lip and / or cleft palate (ICD10 35-37) [28] Cleft lip and palate (CLP) is the most common phenotype, followed by isolated cleft lip (iCL) and isolated cleft palate (iCP). Cleft lip with or without palate (CL+/−P) is used to denote CLP and iCL. Cases were screened to confirm diagnosis and absence of any genetic syndrome or other birth defect by medical practitioners at the mission site. This included pediatricians, nurses, anesthesiologists and surgeons who are all formally licensed, trained and OS certified to work with cleft patients.

Patients were included in the study if they were accompanied by their biological mother (18 years or older), 6 months to 4 years of age, and presented for cleft treatment at the time of the OS mission. Patients were excluded if the child was not the most recent pregnancy, a multiple birth, had a genetic syndrome, or had another co-morbid condition.

### Case recruitment

Cases for the International Family Study (IFS) are recruited on site during OS missions. IFS countries were selected from sites OS identified a priori as having adequate infrastructure to support research and the specific hospital was chosen based on its ability to meet the organization’s ‘Global Standards of Care’. Extensive regional recruitment and community outreach efforts are conducted by OS prior to each mission to assure saturation of the communities. All patients arrive to the mission site to be screened for care over the span of one or two days with all costs covered by OS. The patients are registered and seen by general practitioners, nurses, anesthesiologists, surgeons, and dentists to assess surgical eligibility. Case recruitment for the study occurs at the end of the screening process. Study eligibility criteria were identical for cases in all countries.

### Control definition

Controls were newborns identified from regional neighborhood, clinic, and hospital-based birth centers around the mission site (Table S1 in the **Online Supplementary Document**) whose mother agreed to complete informed consent and the study interview. Individuals were excluded if they had a cleft or any other birth defect, were a multiple birth, or if the mother was younger than 18.

### Control recruitment

Multiple neighborhood, clinic, and hospital-based birth centers were identified prior to each mission by in-country OS partners to represent the catchment area of the OS mission and improve case-control comparability. All maternity wards selected were public to better match demographics of the mission patients. The leadership at the birth center was approached and debriefed on the study, and local authorization was obtained to recruit families along with IRB approval prior to the mission. Each site was visited daily during the mission.

### Data collection

Local volunteers with medical training (ie, nursing/medical students) were identified by OS and underwent training by study members. Local interviewers were used to assure high recruitment and allow completion of the interview in the language of the families; however, the study supervisor was present during all interviews for consistency and to maintain quality. Informed consent was completed before the interview and parents were assured that participation was not required for their child to receive care. Families were interviewed in a private to semi-private area (depending on screening space). Questionnaires have been translated and back translated by certified translators to ensure consistency across countries.

Mother’s interviews took approximately 40 minutes. The interview included questions on family history of cleft, lifestyle and environmental exposures (smoke, alcohol, diet, water source), medical history (parental medical history, use of prescription and nonprescription drugs), demographics (age, pregnancy history, education, employment), and paternal factors (smoke, alcohol, age, employment, education). When the father was present, a limited interview is independently completed on medical history, environmental, and lifestyle exposures. Our current analysis included data exclusively from the mothers’ interviews.

### Statistical analysis

Descriptive statistics, including proportions for categorical variables and means for continuous variables, were constructed for the child characteristics, parental characteristics, and lifestyle factors. Tests of statistical significance included *t* tests for continuous and χ^2^ tests for categorical variables. Maternal exposure to indoor cook smoke was categorized as a dichotomous variable (yes/no). Maternal smoking was dichotomized (ever/never) for the three months prior to pregnancy and during pregnancy. Smoking pre-pregnancy was not collected in Vietnam, so they are not included in those analyses. Fathers were dichotomized into lifetime smokers (ever) or never smokers and the definition included any type of tobacco product (cigarettes, pipes, chewing tobacco, cigars, other). Household tobacco smoke is defined as any member of the household smoking inside during the mother’s pregnancy with the child (yes/ no). All education variables were harmonized as less than secondary school or secondary school or higher for mothers and fathers separately. Family history of cleft was defined as any first or second degree relative having any cleft. Number of children was classified into 1, 2, or 3 or more.

Logistic regression was used to calculate odds ratios and 95% confidence intervals for smoke exposure (indoor cook smoke, maternal smoking, paternal or household tobacco smoke) and cleft overall and by subtype (iCP, CL+/−P). Models were specified as minimal (adjusted for country, maternal age at the child’s birth, mother’s education, father’s education, and family history of cleft), full (adjusted for minimal model and rural/urban residence and maternal alcohol consumption during pregnancy), and a mutually adjusted model including all previous covariates and mutual adjustment for all smoke variables. Additional adjustment for demographics of the child and parent (eg, child’s sex, maternal employment, paternal employment, paternal age at child’s birth), environmental and lifestyle factors (water source, folic acid use, and prescription/ nonprescription drug use) were considered as potential confounders but were not included in the final model as the measures of association did not meaningfully change (difference in effect <10%). Heterogeneity of effects by country was investigated by including interaction terms for exposures of interest and by stratification. Missing values were handled by exclusion as they were generally low (<10%).

Secondary analyses were conducted to assess if the findings differed by cleft subtype (iCP and CL+/−P) as they are often considered to have different etiologies. Heterogeneity of the indoor cook smoke finding by country was evaluated using stratified analysis and by excluding country data one at a time. To evaluate the independent effect of cook smoke on cleft, without confounding by maternal smoking, we repeated the analysis restricted to never smoking mothers. Additional sensitivity analyses were done by maternal education, paternal education, income level, and age of cases (limited to less than one year). Income was only available for Vietnam, the Philippines and Morocco due to cultural sensitivity and medical mission considerations. When available, income quartile groups were defined by country based off of the income level reported by controls. All analyses were completed using SAS 9.4 (SAS Institute Inc., Cary, USA) and R (R Foundation for Statistical Computing, Vienna, Austria).

## RESULTS

A total of 4426 eligible children were identified from the 7 countries between 2012 and 2017. Of these: 58 participants were excluded due to missing case status and an additional 217 were excluded because they exceeded the newborn to four-year inclusion criteria. 4151 participants were included in the final data set: 2137 cases (51%) and 2014 controls (49%) with the majority coming from Vietnam (31.8%), followed by the Philippines (22.3%), Honduras (22.1%), Congo (10.2%), Madagascar (5.1%), Morocco (4.3%), and Nicaragua (4.2%). Cases and controls were recruited simultaneously in all years the study was active with the exception of a delay in control collection in Vietnam in 2012 due to approval delays (Table S2 in the **Online Supplementary Document**). The case phenotype distribution consisted of 1198 (56.1%) with CLP, 553 (25.9%) with iCL, and 306 (14.3%) with iCP (**Table 1**).

**Table 1:** Child characteristics of case and control from all countries (N = 4151)

	Cases (N = 2137)								Controls (N = 2014)							
	**Total N (%)**	**Congo (142)**	**Honduras (389)**	**Madagascar (128)**	**Morocco (111)**	**Nicaragua (124)**	**Philippines (573)**	**Vietnam (670)**	**Total N (%)**	**Congo (282)**	**Honduras (528)**	**Madagascar (85)**	**Morocco (69)**	**Nicaragua (47)**	**Philippines (353)**	**Vietnam (650)**
Child's cleft type:*																
Cleft lip and palate (CLP)	1198 (56.1%)	59 (41.5%)	228 (58.6%)	79 (61.7%)	63 (56.8%)	89 (71.8%)	294 (51.3%)	386 (57.6%)	–	–	–	–	–	–	–	–
Cleft lip only (iCL)	553 (25.9%)	72 (50.7%)	81 (20.8%)	34 (26.6%)	34 (30.6%)	22 (17.7%)	157 (27.4%)	153 (22.8%)	–	–	–	–	–	–	–	–
Cleft palate only (iCP)	306 (14.3%)	10 (7.0%)	68 (17.5%)	15 (11.7%)	11 (9.9%)	10 (8.1%)	67 (11.7%)	125 (18.7%)	–	–	–	–	–	–	–	–
**Child's gender: †**																
Male	1248 (58.4%)	58 (40.8%)	227 (58.4%)	69 (53.9%)	71 (64.0%)	74 (59.7%)	360 (62.8%)	389 (58.1%)	1083 (53.8%)	104 (36.9%)	264 (50.0%)	35 (41.2%)	39 (56.5%)	29 (61.7%)	253 (71.7%)	359 (55.2%)
Female	854 (40.0%)	51 (35.9%)	162 (41.6%)	59 (46.1%)	40 (36.0%)	50 (40.3%)	213 (37.2%)	279 (41.6%)	835 (41.5%)	86 (30.5%)	264 (50.0%)	50 (58.8%)	30 (43.5%)	18 (38.3%)	99 (28.0%)	288 (44.3%)
**Birth order: ‡**																
First Child	708 (33.1%)	35 (24.6%)	124 (31.9%)	36 (28.1%)	30 (27.0%)	56 (45.2%)	177 (30.9%)	250 (37.3%)	799 (39.7%)	98 (34.8%)	227 (43.0%)	37 (43.5%)	23 (33.3%)	19 (40.4%)	117 (33.1%)	278 (42.8%)
Second Child	717 (33.6%)	31 (21.8%)	116 (29.8%)	42 (32.8%)	41 (36.9%)	36 (29.0%)	156 (27.2%)	295 (44.0%)	705 (35.0%)	65 (23.0%)	175 (33.1%)	22 (25.9%)	23 (33.3%)	19 (40.4%)	103 (29.2%)	298 (45.8%)
Third or later	700 (32.8%)	74 (52.1%)	147 (37.8%)	49 (38.3%)	40 (36.0%)	32 (25.8%)	235 (41.0%)	123 (18.4%)	503 (25.0%)	119 (42.2%)	124 (23.5%)	26 (30.6%)	23 (33.3%)	9 (19.1%)	133 (37.7%)	69 (10.6%)

*Missing by country (n cases): Congo (1), Honduras (12), Madagascar (0), Morocco (3), Nicaragua (3), Philippines (55), Vietnam (6).

† Missing by country (n cases, n controls): Congo (33, 92), Honduras (0,0), Madagascar (0,0), Morocco (0,0), Nicaragua (0,0), Philippines (0,1), Vietnam (2,3).

‡ Missing by country (n cases, n controls): Congo (2,0), Honduras (2,2), Madagascar (1,0), Morocco (0,0), Nicaragua (0,0), Philippines (5,0), Vietnam (2,5).

Characteristics of the study population are described in **Table 1**, **Table 2** and **Table 3**. Case mothers were on average six months older than controls (*P* = 0.008) and less often employed (*P* = 0.03). Control mothers (81.1% vs 66.4%) and fathers (78.2% vs 64.2%; both *P* < 0.001) were more likely to have a secondary education. No difference was observed in father’s age, father’s employment status, or maternal smoking (prior to or during pregnancy). A higher proportion of cases reported cooking indoors over an open flame (58.3% vs 36.1%). Fewer case mothers reported drinking alcohol pre-pregnancy (8.5% vs 12.4%; *P* < 0.001); however, they were more likely to report drinking during pregnancy (*P* = 0.09), living in a rural area, smoking in the household, and that the father of the child smoked (all *P* < 0.001). The distribution of all five smoking variables significantly differed across countries (all *P* < 0.05).

**Table 2:** Parental characteristics of cases and controls from all countries (N = 4151)

	Cases (N = 2137)								Controls (N = 2014)							
	**Total N (%)**	**Congo (142)**	**Honduras (389)**	**Madagascar (128)**	**Morocco (111)**	**Nicaragua (124)**	**Philippines (573)**	**Vietnam (670)**	**Total N (%)**	**Congo (282)**	**Honduras (528)**	**Madagascar (85)**	**Morocco (69)**	**Nicaragua (47)**	**Philippines (353)**	**Vietnam (650)**
**Maternal age at birth** Mean (std) *	27.3 (6.36)	28.0 (6.29)	26.3 (6.61)	26.2 (5.94)	27.9 (5.84)	25.0 (6.68)	27.8 (7.02)	27.7 (5.49)	26.8 (5.85)	27.6 (5.72)	24.5 (5.40)	26.0 (6.46)	29.5 (6.47)	24.5 (6.10)	26.7 (6.17)	28.4 (5.12)
**Paternal age at birth ** **Mean (std) †**	30.1 (6.40)	33.6 (6.79)	29.2 (6.78)	29.3 (5.97)	33.8 (5.72)	27.7 (6.29)	29.9 (6.81)	30.4 (5.47)	30.0 (6.37)	33.7 (5.90)	27.7 (6.26)	28.6 (5.68)	34.9 (5.96)	27.6 (6.89)	29.0 (6.70)	31.4 (5.25)
**Education level – Mother: ‡**																
Primary school or less	692 (32.4%)	44 (31.0%)	225 (57.8%)	55 (43.0%)	81 (73.0%)	59 (47.6%)	91 (15.9%)	137 (20.4%)	353 (17.5%)	22 (7.8%)	162 (30.7%)	21 (24.7%)	47 (68.1%)	9 (19.1%)	37 (10.5%)	55 (8.5%)
Secondary or more	1420 (66.4%)	96 (67.6%)	162 (41.6%)	71 (55.5%)	30 (27.0%)	63 (50.8%)	475 (82.9%)	523 (78.1%)	1633 (81.1%)	259 (91.8%)	365 (69.1%)	64 (75.3%)	22 (31.9%)	38 (80.9%)	314 (89.0%)	571 (87.8%)
**Education level – Father:** §																
Primary school or less	587 (27.5%)	15 (10.6%)	189 (48.6%)	47 (36.7%)	50 (45.0%)	47 (37.9%)	112 (19.5%)	127 (19.0%)	319 (15.8%)	6 (2.1%)	165 (31.2%)	17 (20.0%)	26 (37.7%)	11 (23.4%)	53 (15.0%)	41 (6.3%)
Secondary or more	1371 (64.2%)	115 (81.0%)	152 (39.1%)	62 (48.4%)	36 (32.4%)	55 (44.4%)	449 (78.4%)	502 (74.9%)	1575 (78.2%)	266 (94.3%)	331 (62.7%)	60 (70.6%)	31 (44.9%)	33 (70.2%)	296 (83.9%)	558 (85.8%)
**Family history of cleft: ‖**																
Yes	649 (30.4%)	16 (11.3%)	117 (30.1%)	39 (30.5%)	22 (19.8%)	44 (35.5%)	319 (55.7%)	92 (13.7%)	121 (6.0%)	8 (2.8%)	49 (9.3%)	5 (5.9%)	2 (2.9%)	4 (8.5%)	35 (9.9%)	18 (2.8%)
Folic acid: **																
Yes	854 (40.0%)	83 (58.5%)	356 (91.5%)	61 (47.7%)	5 (4.5%)	91 (73.4%)	209 (36.5%)	49 (7.3%)	975 (48.4%)	220 (78.0%)	488 (92.4%)	70 (82.4%)	2 (2.9%)	30 (63.8%)	109 (30.9%)	56 (8.6%)
**Smoking pre-pregnancy – Mother:** ††																
Yes	39 (1.8%)	1 (0.7%)	10 (2.6%)	0 (0%)	0 (0%)	5 (4.0%)	18 (3.1%)	NA	39 (1.9%)	0 (0%)	11 (2.1%)	1 (1.2%)	1 (1.4%)	3 (6.4%)	21 (5.9%)	NA
**Smoking during pregnancy – Mother:** ‡‡																
Yes	15 (0.7%)	0 (0%)	4 (1.0%)	0 (0%)	1 (0.9%)	3 (2.4%)	7 (1.2%)	5 (0.7%)	18 (0.9%)	0 (0%)	6 (1.1%)	0 (0%)	1 (1.4%)	1 (2.1%)	10 (2.8%)	2 (0.3%)
**Smoking – Father:** §§																
Yes	953 (44.6%)	31 (21.8%)	113 (29.0%)	42 (32.8%)	30 (27.0%)	48 (38.7%)	351 (61.3%)	338 (50.4%)	736 (36.5%)	40 (14.2%)	130 (24.6%)	28 (32.9%)	22 (31.9%)	14 (29.8%)	197 (55.8%)	305 (46.9%)
**Alcohol pre-pregnancy – Mother:**‖‖																
Yes	182 (8.5%)	37 (26.1%)	25 (6.4%)	14 (10.9%)	0 (0%)	0 (0%)	72 (12.6%)	34 (5.1%)	249 (12.4%)	86 (30.5%)	45 (8.5%)	10 (11.8%)	1 (1.4%)	0 (0%)	58 (16.4%)	49 (7.5%)
Alcohol during pregnancy – Mother: ***																
Yes	115 (5.4%)	36 (25.4%)	9 (2.3%)	13 (10.2%)	1 (0.9%)	2 (1.6%)	29 (5.1%)	25 (3.7%)	135 (6.7%)	75 (26.6%)	15 (2.8%)	10 (11.8%)	0 (0%)	2 (4.3%)	11 (3.1%)	22 (3.4%)

std – standar deviation

*Missing by country (n cases, n controls): Congo (16,9), Honduras (12,12), Madagascar (4,1), Morocco (7,5), Nicaragua (9,0), Philippines (9,4), Vietnam (23,83)

†Missing by country (n cases, n controls): Congo (48,83), Honduras (48,26), Madagascar (12,10), Morocco (21,17), Nicaragua (48,4), Philippines (45,16), Vietnam (59,130).

‡Missing by country (n cases, n controls): Congo (2,1), Honduras (2,1), Madagascar (2,0), Morocco (0,0), Nicaragua (2,0), Philippines (7,2), Vietnam (10,24).

§Missing by country (n cases, n controls): Congo (12,10), Honduras (48,32), Madagascar (19,8), Morocco (25,12), Nicaragua (22,3), Philippines (12,4), Vietnam (41,51).

‖Missing by country (n cases, n controls): Congo (19,49), Honduras (36,45), Madagascar (15,9), Morocco (5,2), Nicaragua (6,5), Philippines (73,102), Vietnam (79,.44).

**Missing unknown due to question formatting.

††Missing by country (n cases, n controls): Congo (5,9), Honduras (6,3), Madagascar (5,2), Morocco (7,2), Nicaragua (3,10), Philippines (15,4), Vietnam (NA, NA).

‡‡ Missing by country (n cases, n controls): Congo (95,172), Honduras (21,13), Madagascar (11,2), Morocco (5,2), Nicaragua (16,11), Philippines (15,5), Vietnam (31,7).

§§ Missing by country (n cases, n controls): Congo (5,7), Honduras (12,17), Madagascar (13,10), Morocco (2,1), Nicaragua (8,3), Philippines (30,40), Vietnam (25,34).

‖‖Missing by country (n cases, n controls): Congo (3,3), Honduras (5,2), Madagascar (5,0), Morocco (2,0), Nicaragua (124,47), Philippines (19,5), Vietnam (21,9).

***Missing by country (n cases, n controls): Congo (4,6), Honduras (21,10), Madagascar (6,0), Morocco (5,2), Nicaragua (19,3), Philippines (58,94), Vietnam (28,13).

**Table 3:** Lifestyle factors of cases and controls from all countries (N = 4151)

	Cases (N = 2137)									Controls (N = 2014)						
	Total N (%)	**Congo (142)**	**Honduras (389)**	**Madagascar (128)**	**Morocco (111)**	**Nicaragua (124)**	**Philippines (573)**	**Vietnam (670)**	**Total N (%)**	**Congo (282)**	**Honduras (528)**	**Madagascar (85)**	**Morocco (69)**	**Nicaragua (47)**	**Philippines (353)**	**Vietnam (650)**	
Location:*
Rural	1178 (55.1%)	42 (29.6%)	208 (53.5%)	80 (62.5%)	42 (37.8%)	67 (54.0%)	278 (48.5%)	461 (68.8%)	604 (30.0%)	23 (8.2%)	166 (31.4%)	25 (29.4%)	18 (26.1%)	6 (12.8%)	116 (32.9%)	250 (38.5%)	
Urban	814 (38.1%)	87 (61.3%)	159 (40.9%)	45 (35.2%)	54 (48.6%)	51 (41.1%)	263 (45.9%)	155 (23.1%)	1178 (58.5%)	166 (58.9%)	324 (61.4%)	59 (69.4%)	42 (60.9%)	28 (59.6%)	204 (57.8%)	355 (54.6%)	
**Smoking in the household during pregnancy:†**
Yes	941 (44.0%)	30 (21.1%)	94 (24.2%)	53 (41.4%)	23 (20.7%)	41 (33.1%)	307 (53.6%)	393 (58.7%)	725 (36.0%)	29 (10.3%)	120 (22.7%)	44 (51.8%)	15 (21.7%)	16 (34.0%)	157 (44.5%)	344 (52.9%)	
**Cooking indoors:‡**
Yes	1246 (58.3%)	51 (35.9%)	241 (62.0%)	117 (91.4%)	41 (36.9%)	68 (54.8%)	386 (67.4%)	342 (51.0%)	728 (36.1%)	54 (19.1%)	214 (40.5%)	83 (97.6%)	19 (27.5%)	20 (42.6%)	160 (45.3%)	178 (27.4%)	
**Water source before/ during pregnancy:**§
Well water	776 (36.3%)	19 (13.4%)	122 (31.4%)	48 (37.5%)	15 (13.5%)	40 (32.3%)	167 (29.1%)	365 (54.5%)	447 (22.2%)	4 (1.4%)	104 (19.7%)	32 (37.6%)	7 (10.1%)	7 (14.9%)	57 (16.1%)	236 (36.3%)	
Public water	622 (29.1%)	102(71.8%)	100 (25.7%)	40 (31.2%)	83 (74.8%)	52 (41.9%)	127 (22.2%)	118 (17.6%)	816 (40.5%)	242 (85.8%)	119 (22.5%)	44 (51.8%)	44 (63.8%)	28 (59.6%)	89 (25.2%)	250 (38.5%)	
River/ lake water	83 (3.9%)	5 (3.5%)	0 (0%)	2 (1.6%)	0 (0%)	0 (0%)	32 (5.6%)	44 (6.6%)	22 (1.1%)	1 (0.4%)	0 (0%)	0 (0%)	2 (2.9%)	0 (0%)	12 (3.4%)	7 (1.1%)	

*Missing by country (n cases, n controls): Congo (13,93), Honduras (22,38), Madagascar (3,1), Morocco (15,9), Nicaragua (6,13), Philippines (32,33), Vietnam (54,45).

†Missing by country (n cases, n controls): Congo (3,7), Honduras (1,5), Madagascar (2,0), Morocco (5,2), Nicaragua (1,9), Philippines (21,22), Vietnam (7,5).

‡Missing by country (n cases, n controls): Congo (2,6), Honduras (2,7), Madagascar (3,0), Morocco (2,2), Nicaragua (1,10), Philippines (36,48), Vietnam (13,8).

§Percentages will not add up to 100 because the question allowed multiple responses.

The relationship between smoke exposures and the odds of all cleft is shown in **Table 4**. A strong positive association was found between cooking indoors over an open flame and risk of all cleft types. Mothers who reported cooking over an open flame indoors were 49% more likely to have a child with a cleft after adjusting for country, maternal age, mother and father education, family history of cleft, rural/ urban residence, alcohol consumption during pregnancy and all smoke variables [Model 3]. With respect to maternal smoking during pregnancy, the odds ratio (OR) was elevated but the confidence interval included the null (OR = 1.65, 95% confidence interval (CI) = 0.5, 5.6). The prevalence of mothers who smoked prior to or during pregnancy was low (prior: 39 cases (1.8%) and 39 controls (1.9%); during: 15 cases (0.7%) and 18 controls (0.9%)). No association was found with smoking the three months prior to pregnancy or exposure to household tobacco smoke. There was a positive association with ever paternal smoking and cleft (OR = 1.18, 95% CI = 0.96,1.5) however the confidence interval included the null. The results of **Table 4** were nearly identical when restricted to women with no history of smoking (n = 4057). No evidence of interaction by country was found (all *P* > 0.05).

**Table 4:** Adjusted odds ratios (OR) of smoke related factors and cleft lip and/or palate in all countries (N = 4151)

	Model 1*			Model 2 †			Model 3 ‡		
	**OR**	**95% CI**	***P*-value**	**OR**	**95% CI**	***P*-value**	**OR**	**95% CI**	***P*-value**

Cooking indoors over open flame	1.93	(1.64, 2.27)	<.0001	1.51	(1.26, 1.81)	<.0001	1.49	(1.23, 1.79)	<.0001
Smoking Pre-pregnancy- Mother	0.88	(0.49, 1.58)	0.67	0.92	(0.49, 1.70)	0.78	1.65	(0.50, 5.61)	0.52
Smoking during pregnancy- Mother	1.20	(0.45, 3.34)	0.72	1.39	(0.49, 4.05)	0.54	0.79	(0.38, 1.61)	0.41
Smoking- Father	1.11	(0.95, 1.30)	0.19	1.10	(0.93, 1.31)	0.28	1.18	(0.96, 1.47)	0.12
Smoking in the household during pregnancy	1.04	(0.89, 1.23)	0.61	0.98	(0.83, 1.17)	0.86	0.85	(0.68, 1.06)	0.14

OR – odds ratio, CI – confidence interval

*Model 1 – Adjusted for country, maternal age, mother and father education (primary or less/ secondary or more), family history of cleft.

†Model 2 – Additionally adjusted for rural/ urban home and alcohol consumption during pregnancy.

‡Model 3 – Mutually adjusted for all smoke variables and Model 2 covariates.

The analyses restricting to CL+/−P (cases = 1751; excluding iCP) are explored in **Table 5**. Cooking indoors over an open flame was associated with a modest increase in risk of cleft (OR = 1.55, 95% CI = 1.3, 1.9) compared to the full case set. For iCP (cases = 306), only cooking indoors over an open flame in the minimally adjusted model showed elevated risk (OR = 1.65, 95% CI = 1.2, 2.3) (Table S2 in the **Online Supplementary Document**). No other smoking variables were associated with iCP in any model.

**Table 5:** Adjusted odds ratios (OR) of smoke related factors and cleft lip with or without cleft palate (excluding iCP) in all countries (N = 3765)

	Model 1*			Model 2 †			Model 3 ‡		
	**OR**	**95% CI**	***P*-value**	**OR**	**95% CI**	***P*-value**	**OR**	**95% CI**	***P*-value**
Cooking indoors over open flame	2.02	(1.70, 2.41)	<.0001	1.56	(1.29, 1.90)	<.0001	1.55	(1.27, 1.89)	<.0001
Smoking Pre-pregnancy- Mother	0.91	(0.50, 1.66)	0.76	0.95	(0.50, 1.80)	0.87	2.08	(0.61, 7.30)	0.43
Smoking during pregnancy- Mother	1.43	(0.53, 4.02)	0.48	1.65	(0.57, 4.86)	0.35	0.74	(0.34, 1.57)	0.24
Smoking- Father	1.08	(0.92, 1.28)	0.35	1.08	(0.90, 1.29)	0.42	1.14	(0.91, 1.43)	0.26
Smoking in the household during pregnancy	1.06	(0.89, 1.25)	0.54	1.00	(0.83, 1.20)	0.98	0.87	(0.69, 1.10)	0.25

CI – confidence interval, OR – odds ratio, iCP – cleft palate only

*Model 1 – Adjusted for country, maternal age, mother and father education (primary or less/ secondary or more), family history of cleft.

†Model 2 – Additionally adjusted for rural/ urban home and alcohol consumption during pregnancy.

‡Model 3 – Mutually adjusted for all smoke variables and Model 2 covariates.

The impact of rural/ urban residence, parental education, and case age on risk of all cleft types was explored in stratified analyses. Cooking indoors over an open flame was associated with cleft in both rural and urban residence. However, the magnitude was higher for urban (OR = 1.71, 95% CI = 1.3,2.3) than rural (OR = 1.28, 95% CI = 1.0, 1.7) (data not shown). Sensitivity analyses showed that the results were as strong by education group for both parents (less than secondary vs secondary or higher) using stratification, by paternal smoking status (yes v. no) using stratification, after adjusting for income, and when restricting to cases under one year of age (group most comparable to newborn controls).

**Figure 1:**
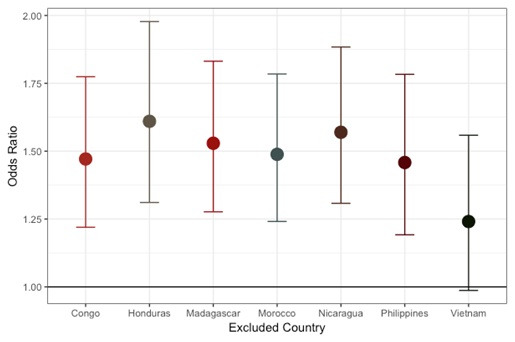
Cooking indoors over an open flame – odds ratio and 95% confidence interval excluding each country (all cleft types combined).

**Figure 2:**
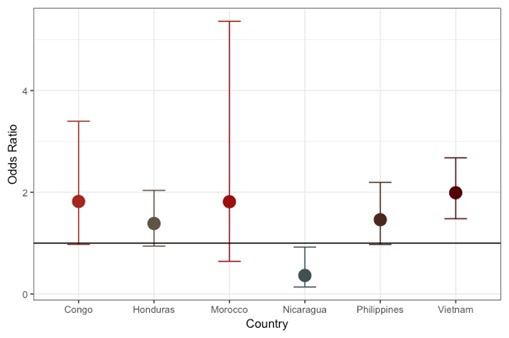
Cooking indoors over an open flame – odds ratio and 95% confidence interval by country (all cleft types combined).

Effect estimates after exclusion of individual countries are shown in **Figure 1**. The overall effect was not dramatically influenced by data from any single country with the exception of Vietnam. The association was slightly reduced (OR = 1.25 (1.0,1.6)) with the removal of Vietnam, which contributed 31.8% of the data set. The individual association of cooking indoors over an open flame by country is shown in **Figure 2**. Positive associations were found in every country besides Nicaragua. The most extreme results were observed in Vietnam (OR = 2.05, 95% CI = 1.5, 2.8) followed by the Congo (OR = 1.82, 95% CI = 1.0, 3.4), and the Philippines (OR = 1.47, 95% CI = 1.0,2.2). Madagascar did not contribute to the figures due to minimal variation (93.9% reported cooking indoors over an open flame). The results observed for CL+/−P were comparable and slightly further from the null with Congo, Honduras and the Philippines reaching statistical significance (Figure S1 and S2 in the **Online Supplementary Document**). The iCP results showed a generally positive association with cooking indoors over an open flame (Figure S3 and S4 in the **Online Supplementary Document**).

## DISCUSSION

This is the first study to report on the association between cooking indoors over an open flame and non-syndromic cleft using data from multiple low-resource countries. Mothers who reported indoor cook smoke exposure were approximately 50% more likely to have a child with a cleft. This result was present after controlling for suspected confounders (including other sources of smoke exposure) and in countries with different socioeconomic characteristics and access to care, as well as being consistent for 5 of 7 countries (Vietnam, the Philippines, Honduras, DRC, and Morocco). The negative association found in Nicaragua was underpowered and will need to be explored further as the sample size increases. No association was found with maternal smoking (prior to or during pregnancy), paternal smoking (lifetime), environmental tobacco smoke, or folic acid supplementation.

Similar to our findings, a prior case-control study by Liu et al. in China found that indoor air pollution was associated with an increased odds of cleft if the house was not ventilated (OR = 4.5, 95% CI = 1.6-12.9)) and attributed this to coal-burning heating sources [9]. A second study in the Congo found the odds of cleft was 6-times higher among mothers who reported cooking indoors [10]. However, the sample size was small (n = 162 cases; 162 matched controls) and they didn’t adjust for additional confounding factors beyond matching. Notably, they also found no maternal smoking effect but did find a positive association with paternal smoking.

Although we did not observe an effect of mother’s smoking on the risk of cleft, the frequency of maternal smoking was very low, and smoking is most often not economically accessible or culturally accepted for women in low-resource populations. According to 2016 World Bank data, 1% of women smoke in Vietnam, 7.8% in the Philippines, 2% in Honduras, and 0.80% in Morocco [29]. Although the US Surgeon General report identified maternal smoking during pregnancy as a risk factor for cleft [30], it is unclear if these findings are generalizable to low-resource settings. The literature consistently supports an association between maternal smoking and risk of cleft. Specifically, a meta-analysis of 23 case-control and 6 cohort studies found that mothers who smoked were 37% more likely to have a child with CL+/−P than never smokers [12,14,31]. It is worth noting that 27 of 29 studies were conducted in populations of European decent and developed countries, which may reflect a different risk profile than the individuals in our study.

The effects of paternal smoking and ETS on cleft is less conclusive. Paternal smoking was evaluated in a subset of the current data from Vietnam, the Philippines, Morocco, and Honduras (n = 626 father/child duos) and no association with cleft was found [32]. Studies in Norway [33], India [34], and China [35] have found that exposure to ETS, defined as an exposure to passive tobacco smoke during the first trimester at home or work, is associated with an increased risk of cleft (OR = 1.6, 95% CI = 1.0-2.5; OR = 2.0, 95% CI = 1.2-3.4; OR = 2.46, 95% CI = 0.99-6.08, respectively), however a case-control study based in a large American birth defect registry found no effect [31]. Our study did not see an effect of either paternal smoking or ETS, where ETS is defined as maternal report of passive smoke exposure in the home during pregnancy.

The mechanism for a role of smoke on cleft formation in embryonic development has been described in the current literature. An association between maternal periconceptional exposure to secondhand tobacco smoke and cleft in offspring has been consistently found [36-41] in epidemiological studies, while 2 studies have linked clefting with maternal exposure to other indoor air pollutants and combustion byproducts [9,42]. The incomplete combustion of tobacco and other organic compounds, including fuels for cooking and heating, produces numerous airborne chemicals. Secondhand smoke from tobacco and smoke from fuel combustion contain known teratogens, including polycyclic aromatic hydrocarbons (PAHs), carbon monoxide (CO), and heavy metals. Shum et al. demonstrated that periconceptional maternal exposure to the PAH benzo[a]pyrene causes cleft in genetically “nonresponsive” inbred mice, which were metabolically deficient [43]. Maternal exposure to low levels of CO has been shown to cause tissue hypoxia in rat fetuses [44], which diminishes cellular metabolism of benzo[a]pyrene [45], suggesting a potential role of CO in cleft development. Combustion of both tobacco and biomass fuels emit heavy metals, including cadmium, which has been shown to cause cleft development in rats [46,47]. At the human population level, Langlois et al. showed that maternal occupational exposure in work environments with greater levels of PAHs was associated with greater odds of cleft [42].

Bias due to control selection is a concern common to all case-control studies. Selection of more affluent controls could influence the representativeness of cooking methods in the sample with respect to the underlying base population. The replication of the association across sites (**Figure 2**) suggests that selection of non-comparable controls is unlikely to explain the finding due to the variability of SES and access to medical care across countries. In support of this, the association between indoor cook smoke exposure and cleft was present in countries with a variety of surgical care and those where medical missions are the primary care source. Further, differential recruitment by age of cases and controls did not explain the results as the effect magnitude was not diminished when restricting to cases one-year of age and under. The original design restricted to cases age 4 years and under to limit recall bias and in fact approximately 70% of our cases were under one year. Correction for differential selection due to SES was addressed by adjustments for household income and parental education in the analysis. Both the adjusted and stratified models by SES were consistent with the original findings.

A limitation of our study is that we cannot be certain cases are fully population-based. However, regional recruitment efforts were extensive and conducted at least four months prior to each mission. Control samples were collected from women at public neighborhood, clinic, and hospital-based birth centers to limit the oversampling of higher income families. Another concern may be underreporting of smoking or alcohol history by case mothers due to stigma around these behaviors while pregnant. While we did ask information on amount of tobacco products parents used weekly, the data was too sparse and variability too low among mothers to conduct a detailed analysis. Similarly, we did not have data on potential changes in the father’s smoking habits during the pregnancy, which would not adequately classify the fathers smoking status by trimester.

## CONCLUSIONS

Exposure to smoke while cooking is already a well-established health risk in low-resource countries for a wide variety of diseases but has been minimally studied with respect to cleft. We found a 50% increase in cleft risk for mothers reporting cooking over an open flame indoors compared to controls in a diverse group of LMICs. It is necessary to take risk factors specific to low resource settings into account, as those individuals are at the highest risk of being unable to access care and therefore live with the negative health consequences of disease. This information can inform public health interventions and education to potentially prevent disease in populations where care is sparse, and children are most likely to feel the detrimental, lifelong medical and social effects of cleft. Modifiable, patient-centric solutions, such as providing a clean-burning cookstove, will be critical for efforts to decrease the burden of cleft globally and improve lives around the world.

## Additional material

Online Supplementary Document
