# Supplementary material for: The role of smoke from cooking indoors over an open flame and parental smoking on the risk of cleft lip and palate: A case- control study in 7 low-resource countries
Source: J Glob Health. 2020 Aug 14;10(2):020410. doi: 10.7189/jogh.10.020410 (PMC7568926; doi:10.7189/jogh.10.020410)

Table S1. All Sites Used for Case and Control Collection

Country	City	Site
Democratic Republic of Congo	Kinshasa	Operation Smile Democratic Republic of Congo
		Counseil National de l'Orde des Medecins
		Clinique Ngaliema
		Kinshasa General
		Roi Baudouin
		Maternite Kingasani
		El Rapha Clinic
		Kitambo
		Centre De Sante Maternite Lisungi
Honduras	Choluteca	Hospital Regional Del Sur
	Comayagua	Hospital Santa Teresa
	Santa Rosa de Copan	Hospital Regional de Occidente
	Tegucigalpa	Operacion Sonrisa Honduras
		Hospital San Felipe
Madagascar	Antananarivo	Operation Smile Madagascar
		Joseph Ravoahangy Andrianavalona Hospital
	Antsirabe	Vakinankaratra Regional Center Hospital
Morocco	Oujda	Hospital Al Farabi
	Marrakesh	Ibn Tofail Hospital
	Tiznit	Hospital Hasan I
Nicaragua	Managua	Operacion Sonrisa Nicaragua
		Hospital Aleman Nicaraguanese
		Hospital Berta Calderon
Philippines	Bacolod	Our Lady of Mercy Hospital
		Adventist Miller Hospital
	Cauayan City	Cauayan District Hospital
		Isabela United Doctors Medical Center
	Cavite	General Emilio Aguinaldo Memorial Hospital
		Pakamutan ng Dasmarinas
		St. Paul Hospital
	Cebu	Mariquita Young Foundation
		Paanakan Se Mandaue (Mandaue)
		St. Anthony’s Birthing Clinic
		Municipal Health Office of Consolacion
		Daisy’s Birthing Clinic
		University of Cebu Medical Center
		Agnes Birthing Center
	Davao City	Brokenshire Hospital
		Mindanao Cleft Center
	General Santos	General Santos District Hospital
	Iloilo	Qualimed Hospital
		CFC Birthing Clinic
	Pampanga	Ricardo Rodriguez Hospital
		Diosdado Macapagal Hospital
		Jesus A Datu Medical Center
Vietnam	An Giang	An Giang General Hospital
	Dak Lak	Dak Lak District Hospital
	Hanoi	Vietnam Cuba Friendship Hospital
		Operation Smile Vietnam
		Phu San HN (Hanoi Maternity Clinic)
	Ho Chi Minh City	HCMC University Medical Center
		Thu Duc District Hospital
	Hue	Hue University Medical Center

	Quang Ngai	Quang Ngai Hospital

Table S2. Case and Control Breakdown by Year for All Countries

	2012		2013		2014		2015		2016		2017	
	**Control**	**Case**	**Control**	**Case**	**Control**	**Case**	**Control**	**Case**	**Control**	**Case**	**Control**	**Case**
	(n=210)	***(n=136)***	***(n=248)***	***(n=106)***	***(n=419)***	***(n=532)***	***(n=553)***	***(n=493)***	***(n=412)***	***(n=530)***	***(n=172)***	***(n=340)***
**Country**												
**Congo**	117 (55.7%)	50 (36.8%)	91 (36.7%)	33 (31.1%)	0 (0%)	0 (0%)	74 (13.4%)	59 (12.0%)	0 (0%)	0 (0%)	0 (0%)	0 (0%)
**Honduras**	0 (0%)	0 (0%)	0 (0%)	0 (0%)	169 (40.3%)	132 (24.8%)	145 (26.2%)	74 (15.0%)	193 (46.8%)	129 (24.3%)	21 (12.2%)	54 (15.9%)
**Madagascar**	0 (0%)	0 (0%)	0 (0%)	0 (0%)	0 (0%)	0 (0%)	0 (0%)	0 (0%)	85 (20.6%)	128 (24.2%)	0 (0%)	0 (0%)
**Morocco**	0 (0%)	0 (0%)	0 (0%)	0 (0%)	23 (5.5%)	45 (8.5%)	30 (5.4%)	39 (7.9%)	16 (3.9%)	27 (5.1%)	0 (0%)	0 (0%)
**Nicaragua**	0 (0%)	0 (0%)	0 (0%)	0 (0%)	0 (0%)	0 (0%)	0 (0%)	0 (0%)	0 (0%)	0 (0%)	47 (27.3%)	124 (36.5%)
**Philippines**	93 (44.3%)	50 (36.8%)	0 (0%)	0 (0%)	206 (49.2%)	181 (34.0%)	38 (6.9%)	157 (31.8%)	13 (3.2%)	110 (20.8%)	3 (1.7%)	75 (22.1%)
**Vietnam**	0 (0%)	36 (26.5%)	157 (63.3%)	73 (68.9%)	21 (5.0%)	174 (32.7%)	266 (48.1%)	164 (33.3%)	105 (25.5%)	136 (25.7%)	101 (58.7%)	87 (25.6%)

Table S2. Adjusted Odd's Ratios (OR) of Smoke Related Factors and Isolated Cleft Palate in all countries (N= 2320)

	Model 1*			Model 2**			Model 3***		
	OR	95% CI	p-value	OR	95% CI	p-value	OR	95% CI	p-value
Cooking indoors over open flame	1.65	(1.22, 2.23)	0.001	1.33	(0.95, 1.85)	0.10	1.28	(0.91, 1.79)	0.16
Smoking Pre-pregnancy- Mother	0.75	(0.21, 2.10)	0.61	0.82	(0.19, 2.52)	0.76	NA	NA	NA
Smoking during pregnancy- Mother	NA	NA	NA	NA	NA	NA	1.15	(0.26, 3.75)	0.98
Smoking- Father	1.20	(0.90, 1.60)	0.22	1.17	(0.86, 1.59)	0.31	1.35	(0.99, 2.00)	0.14
Smoking in the household during pregnancy	0.99	(0.74, 1.34)	0.97	0.97	(0.71, 1.32)	0.84	0.80	(0.53, 1.19)	0.27

*Model 1-- Adjusted for country, maternal age, mother and father education (primary or less/ secondary or more), family history of cleft
**Model 2-- Additionally adjusted for rural/ urban home and alcohol consumption during pregnancy
***Model 3-- Mutually adjusted for all smoke variables and Model 2 covariates

Figure S1. Cooking Indoors Over an Open Flame- Odds Ratio and 95% CI Excluding each Country (CL+/-P ONLY)

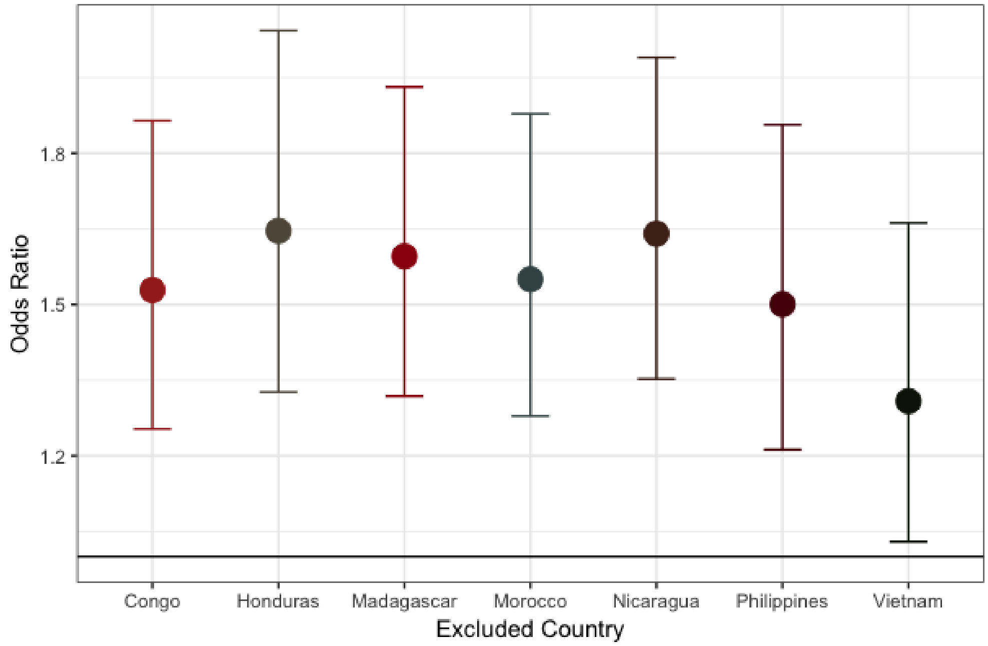

Figure S2. Cooking Indoors Over an Open Flame- Odds Ratio and 95% CI By Country (CL+/-P ONLY)

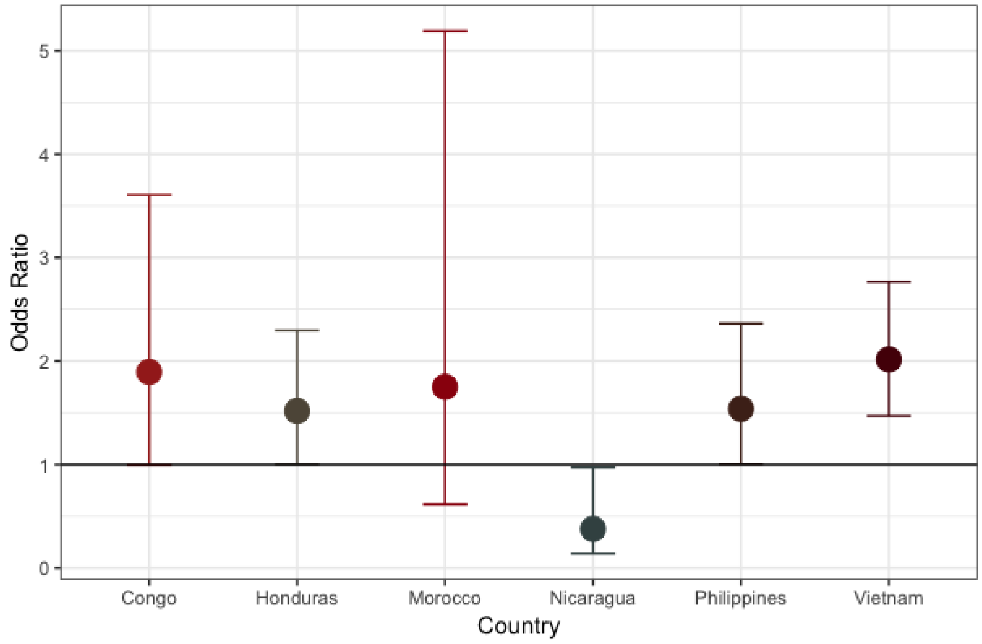

Figure S3. Cooking Indoors Over an Open Flame- Odds Ratio and 95% CI Excluding each Country (iCP ONLY)

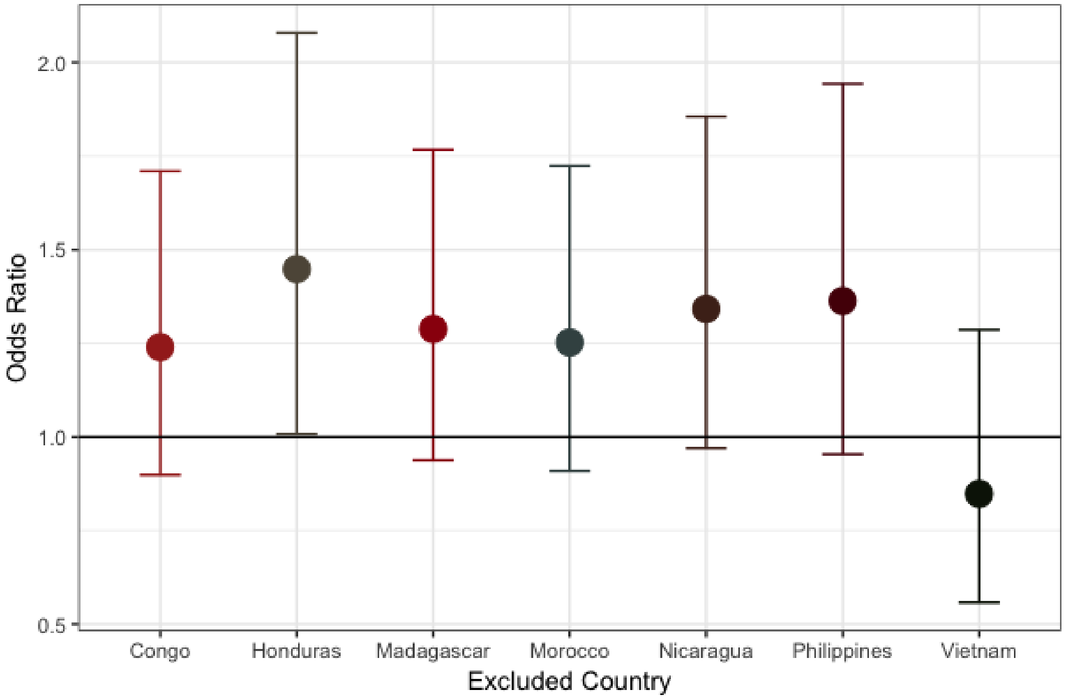

Figure S1. Cooking Indoors Over an Open Flame- Odds Ratio and 95% CI By Country (iCP ONLY)

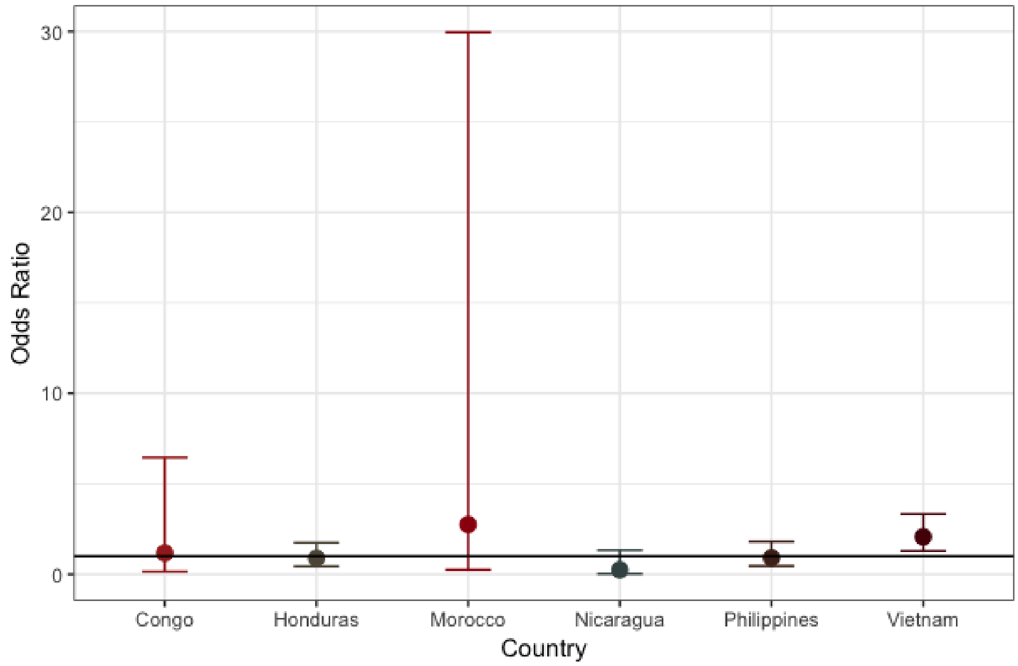

Supplement: Online Supplementary Document [file jogh-10-020410-s001.pdf]
